# The Role of Proinflammatory Cytokines in Temporomandibular Disorders: A Systematic Review

**DOI:** 10.3390/ijms27083677

**Published:** 2026-04-20

**Authors:** Zuzanna Grzech-Leśniak, Agnieszka Matuszewska, Jakub Fiegler-Rudol, Marwan El Mobadder, Rafał Wiench, Mieszko Więckiewicz

**Affiliations:** 1 Department of Experimental Dentistry, Wroclaw Medical University, 50-425 Wroclaw, Poland; zuzanna.grzech-lesniak@student.umw.edu.pl; 2 Department of Pharmacology, Wroclaw Medical University, 50-345 Wroclaw, Poland; agnieszka.matuszewska@umw.edu.pl; 3 Department of Periodontal Diseases and Oral Mucosa Diseases, Faculty of Medical Sciences in Zabrze, Medical University of Silesia, 40-055 Katowice, Poland; s88998@365.sum.edu.pl (J.F.-R.); rwiench@sum.edu.pl (R.W.); 4 Department of Periodontology, Faculty of Dental Medicine, Lebanese University, Beirut P.O. Box 6573/14, Lebanon; marwan.mobader@gmail.com

**Keywords:** temporomandibular disorders, temporomandibular joint, cytokines, TNF-α, IL-1β, IL-6, biomarkers, synovial fluid, orofacial pain, temporomandibular joint degeneration

## Abstract

Temporomandibular disorders (TMDs) are the prevalent causes of orofacial pain and dysfunction of the temporomandibular joint (TMJ) and masticatory muscles. Previous studies have revealed that proinflammatory cytokines play a key role in promoting inflammation, pain, and degeneration within the TMJ. In this context, the present systematic review synthesizes current evidence on various cytokines involved in the pathophysiology of TMDs and evaluates their associations with clinical signs and structural TMJ damage. A PRISMA-guided search (PROSPERO: CRD420251163290) was conducted in PubMed/MEDLINE, Embase, Scopus, and the Cochrane Library to identify human-based, in vivo, and in vitro studies (January 2014 to September 2025) that assessed the roles of proinflammatory cytokines in TMDs. The following data were extracted from the identified studies: cytokine profiles, sampling methods, clinical outcomes, and TMJ structural changes. Study quality and risk of bias were systematically evaluated. A total of 15 studies (clinical, animal, and mechanistic) were included in the review. Tumor necrosis factor-alpha (TNF-α), interleukin-1β (IL-1β), interleukin-6 (IL-6), and interleukin-17 (IL-17) consistently emerged as the major contributors to synovitis, cartilage degradation, nociceptive sensitization, and bone resorption. Human studies showed that high levels of TNF-α, IL-1β, and IL-6 and chemokines such as C-C motif chemokine ligand 2 (CCL2) and regulated on activation, normal T-cell expressed and secreted (RANTES) were associated with TMJ pain, restricted mandibular motion, crepitus, malocclusion, and erosive changes on imaging. An increased ratio of TNF to soluble TNF receptor in synovial fluid correlated with both pain and condylar damage, suggesting that loss of cytokine control contributes to progressive joint destruction. TMDs, particularly inflammatory and degenerative subtypes, are cytokine-driven pathologies rather than purely mechanical disorders. TNF-α, IL-1β, and IL-6 are the promising candidate biomarkers of local inflammation and structural joint pathology. Standardized longitudinal studies are required to validate cytokine-based diagnostics and develop anti-cytokine therapeutics.

## 1. Introduction

### 1.1. Rationale

Temporomandibular disorders (TMDs) are a diverse group of musculoskeletal diseases characterized mainly by the occurrence of pain in the facial and temporal regions, frequently accompanied by limitations of mandibular function [1,2,3,4,5,6,7]. Rather than constituting a single disease entity, TMD is an umbrella term encompassing a range of distinct pathological conditions. The diagnosis and management of TMDs require considerable clinical expertise because of their broad-spectrum and often ambiguous symptoms. Typical manifestations of TMDs include temporomandibular joint (TMJ) pain and/or sounds such as clicking and crepitus, restricted mandibular mobility, discomfort and/or pain in the masticatory muscles, headache, tinnitus, hearing disturbances, and ear pain [8,9,10,11,12]. After chronic lower back pain, TMDs are the second most prevalent musculoskeletal disorder causing pain and disability [13]. Epidemiological studies indicate a high incidence of TMDs in the human population, with prevalences of 9–15% and 3–10% in women and men, respectively [3,14]. This highlights the need for extensive research and increased public health awareness. Although there has been a steady increase in the number of studies on this topic, current efforts remain insufficient. The management of TMDs requires a multidisciplinary approach that integrates dental, neurological, rheumatological, physiotherapeutic, psychological, and oncological perspectives [15]. Treatment strategies comprise three broad categories: (1) conservative interventions such as self-regulation, occlusal splints, and physiotherapy, (2) drug therapy such as anti-inflammatory and immunomodulatory pharmacotherapy and intramuscular and intracapsular injections, and (3) surgical procedures, including total joint replacement [2,3,16]. Beyond clinical manifestations and therapeutic approaches, it is crucial to understand the molecular mechanisms underlying TMDs. Pathological states involving intra-articular degeneration are often associated with elevated levels of proinflammatory cytokines, including interleukin-1β (IL-1β), interleukin-6 (IL-6), and tumor necrosis factor-α (TNF-α), within the synovial fluid (SF). Notably, IL-6 exhibits dual activity, functioning as both proinflammatory and adaptive mediator, while interleukin-11 (IL-11) is involved in adaptive joint responses, and interleukin-10 (IL-10) predominantly exerts anti-inflammatory effects. Moreover, TNF plays a pivotal role in TMJ inflammation through enhancement of its own production and stimulation of downstream mediators, including IL-1, IL-6, and prostaglandins. In contrast, anti-inflammatory cytokines such as interleukin-4 (IL-4) and IL-10, combined with prostaglandin synthesis inhibitors and glucocorticoids, suppress TNF expression [4,5,8,9,17,18,19]. TMJ homeostasis is maintained by dynamic interactions among cytokines, proteolytic enzymes, and other inflammatory mediators [20,21,22,23,24]. 

Emerging evidence suggests the key role of cytokine signaling dysregulation within the TMJ microenvironment in the development and progression of TMD-related pain. Therefore, identifying reliable cytokine biomarkers could enable diagnosis in the early stage, stratification of disease severity, and monitoring of therapeutic response in TMD patients. Although cytokine alteration has been increasingly implicated in TMD, existing evidence remains heterogeneous, with inconsistent findings across studies. Therefore, we conducted a systematic review to synthesize the current knowledge on this topic and clarify the contribution of proinflammatory cytokines to TMD pathophysiology. 

### 1.2. Objectives

This systematic review aimed to evaluate the current evidence regarding the mediating role of proinflammatory cytokines in the pathophysiology of TMDs by synthesizing data from clinical, in vivo, and in vitro studies. The objectives were (1) to identify specific cytokines contributing to inflammation, pain, and tissue degeneration within the TMJ and (2) to determine whether alterations in cytokine profiles correlate with disease severity or clinical presentation. Importantly, although this review discusses cytokine involvement across the spectrum of TMDs, the current body of evidence is predominantly derived from studies on inflammatory and degenerative TMJ conditions. Consequently, the strength of evidence supporting cytokine-mediated mechanisms may vary between different TMD subtypes, and appears to be most robust in joint-related pathologies.

## 2. Materials and Methods

### 2.1. Focused Question (PECO [Population, Exposure, Comparator, Outcome] Framework)

This systematic review was developed based on the PECO framework to address a core research question: compared to normal or lower cytokine levels, whether elevated levels of proinflammatory cytokines are associated with an increased presence or greater severity of TMDs clinical signs and symptoms in individuals with or without TMDs.

The study population included individuals with or without TMDs. The exposure of interest was elevated levels of proinflammatory cytokines, and the comparison group included individuals with normal or lower cytokine levels. The outcome focused on the presence or severity of clinical signs and symptoms of TMDs. This framework allowed us to explore whether inflammatory biomarkers are associated with TMDs pathogenesis and progression.

The overall certainty of evidence in this systematic review was evaluated using the GRADE (Grading of Recommendations, Assessment, Development and Evaluation) framework. The certainty of evidence ranged from low to moderate across the included studies, primarily because of methodological diversity and limitations inherent to observational and experimental designs. 

### 2.2. Search Strategy

This systematic review was conducted in accordance with the Preferred Reporting Items for Systematic Reviews and Meta-Analyses 2020 (PRISMA 2020) guidelines to ensure a transparent and comprehensive reporting process [25]. The review was registered with PROSPERO [registration number: CRD420251163290]. Relevant studies examining the relationship between proinflammatory cytokines and TMDs were identified through a structured literature search across major scientific databases, including PubMed/MEDLINE, Embase, Scopus, and the Cochrane Library. The search covered articles published between January 2014 and September 2025. Two authors (Z.G.-L. and J.F.-R.) conducted independent screening to select articles based on the title, abstract, and inclusion and exclusion criteria for full text. Discrepancies were resolved by consulting two additional authors (M.E.M. and R.W.) until consensus was reached. Additionally, the reference lists of all included articles were manually reviewed to identify further eligible studies. The final analysis included only articles published in English and meeting the predefined inclusion criteria. Table 1 shows the full search strategy used to identify relevant publications.

### 2.3. Eligibility Criteria

Inclusion criteria:

- Human clinical studies (cross-sectional, cohort, and case–control)
- Studies involving animal in vivo models relevant to TMJ inflammation or degeneration
- In vitro mechanistic studies
- Studies evaluating proinflammatory cytokines (e.g., TNF-α, IL-1β, and IL-6)
- English language publications

Exclusion criteria:

- Reviews, conference abstracts, and dissertations
- Non-English publications
- Duplicate or overlapping datasets

### 2.4. Study Selection

To ensure methodological accuracy and minimize selection bias, all retrieved records were independently screened by two authors (Z.G.-L., and J.F.-R.) and a third author was consulted to resolve any disagreements (M.E.M.). Articles were selected by evaluating their full text based on predefined inclusion and exclusion criteria. The following inclusion criteria were considered: (1) studies describing the presence, levels, or roles of proinflammatory cytokines in individuals diagnosed to have TMDs, (2) original research articles, (3) papers published in English language, (4) human studies, including both case–control and observational design, and (5) studies reporting in vitro experiments and animal research. Publications that met predefined quality standards were considered for inclusion (Table 2). Studies were excluded if they were beyond the scope of peer-reviewed literature, including unpublished or non-indexed materials such as dissertations, theses, conference proceedings, and other forms of grey literature. Manuscripts published in language other than English were also omitted. Additionally, duplicate entries and publications sharing the same ethical approval or dataset were removed to avoid redundancy and data overlap. Any discrepancies in the eligibility assessment were resolved through discussion or consultation with a third author. Reference lists of the included articles were also screened to identify additional relevant studies not captured through the initial database search. This systematic approach ensured that the studies most relevant to the review objectives were included in the analysis.

### 2.5. Risk of Bias in Individual Studies and Quality Assessment

Three authors (Z.G.-L., J.F.-R. and M.E.M.) independently assessed the methodological quality of each included study by using a structured appraisal tool tailored to evaluate risk of bias in studies investigating inflammatory biomarkers in TMDs. This assessment followed a predefined checklist comprising nine core criteria, outlined in Table 2. Each criterion was rated on a binary scale: 1 point if the criterion was clearly met; 0 point if the criterion remained unmet or if there was ambiguity. Total scores ranged from 0 to 9, with studies classified to have high (0–3), moderate (4–6), or low (7–9) risk of bias. To ensure consistency and objectivity, scoring discrepancies were resolved through discussion, and a fourth author was consulted if consensus could not be reached. The assessment was based on methodological standards from the Cochrane Handbook for Systematic Reviews of Interventions. No studies were excluded solely because of a high risk of bias. The following domains were evaluated in the quality checklist:

(A) Clear description of the proinflammatory markers assessed (e.g., IL-6, TNF-α, and CRP);

(B) Appropriateness and clarity of biomarker collection and analysis protocols;

(C) Inclusion and definition of relevant comparison groups or control populations;

(D) Validity and reliability of outcome measures related to TMDs diagnosis or symptom severity;

(E) Detailed reporting of participant selection criteria and demographics;

(F) Justification of sample size or power analysis;

(G) Use of appropriate statistical methods with transparent reporting of findings;

(H) Comprehensive outcome reporting with no selective data omission; and

(I) Full disclosure of funding sources and potential conflicts of interest.

### 2.6. Data Extraction

After the eligible studies were selected, three authors (Z.G.-L., J.F.-R. and M.E.M.) independently extracted data using a structured process to ensure data completeness and consistency. A standardized form was used to capture all relevant information from each article, including bibliographic details (author, year of publication, and country), study design (case–control and cross-sectional), characteristics of the study population (sample size, age, sex distribution, and diagnostic criteria for TMDs), and methods used for cytokine detection (enzyme-linked immunosorbent assay (ELISA), polymerase chain reaction (PCR), and multiplex assays). Data were also collected on specific proinflammatory cytokines investigated, such as IL-1β, IL-6, and TNF-α, and their reported concentrations in TMDs vs. control groups. Whenever applicable, the anatomical sampling site (e.g., synovial fluid (SF), blood, and saliva), assay sensitivity, and statistical significance of group differences were recorded. Additionally, details regarding confounding variables, subgroup analyses, and associations with clinical parameters (e.g., pain intensity and joint function) were documented to provide context for interpretation.

Discrepancies between the three authors during the data extraction process were resolved through discussion, with input from a fourth author whenever required. This rigorous approach ensured that data regarding key variables were consistently retrieved from different studies, forming a reliable basis for the synthesis and comparison of findings.

### 2.7. Study Selection Results

**Figure 1:**
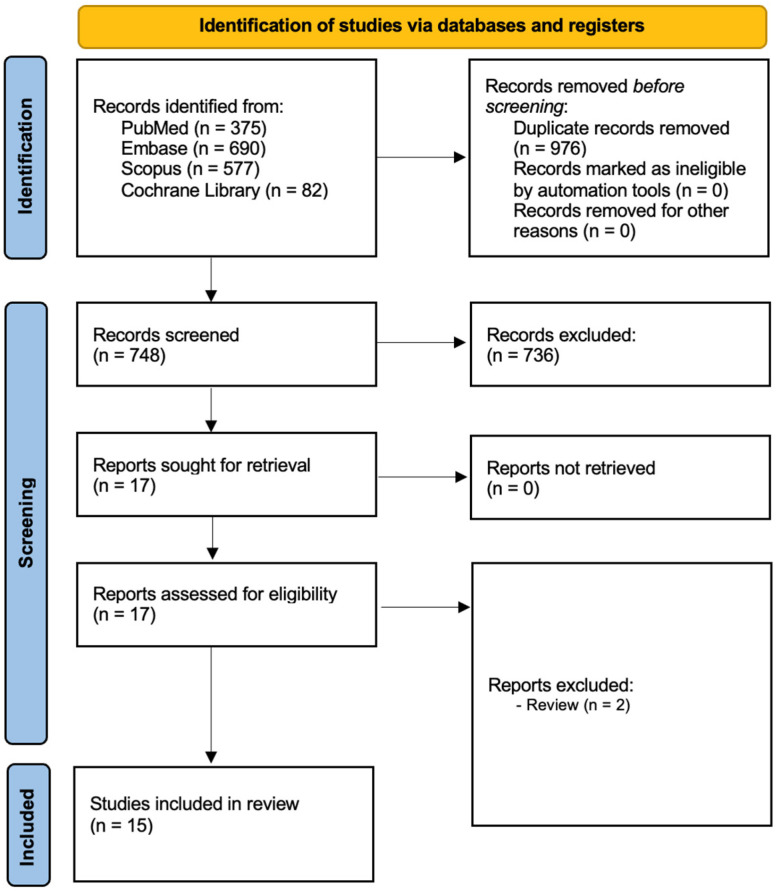
Prisma 2020 flow diagram for study identification, inclusion, and exclusion.

A total of 1724 records were found across four databases: PubMed (375), Embase (690), Scopus (577), and Cochrane (82) (Figure 1). After eliminating 976 duplicates, 748 records remained for screening. Of these, 736 records were excluded, and 17 records were retrieved for further review. Based on the eligibility criteria, 2 records were excluded because they were reviews. Finally, 15 studies were included in the qualitative analysis.

## 3. Results

### 3.1. Quality Appraisal of the Included Studies

Table 2 shows the risk of bias for the included studies. All studies provided a clear description of the proinflammatory markers assessed, appropriateness and clarity of biomarker collection and analysis protocols, validity and reliability of outcome measures related to TMD diagnosis or symptom severity, detailed reporting of participant selection criteria and demographics, use of appropriate statistical methods with transparent reporting of findings, and comprehensive outcome reporting with no selective data omission. The most challenging criterion, applicable for the smallest proportion of studies, was justification of sample size or power analysis, which was identified in only five articles [9,17,18,22,23]. The article with the lowest score was Dozet et al. [8], which received 7 points. Although the included studies exhibited heterogeneity in design and methodology, all met the majority of the predefined quality criteria within the applied assessment framework. Key domains, including patient selection, outcome definition, and methodological transparency, were consistently adequately addressed. As a result, none of the studies met the thresholds for moderate or high risk of bias, supporting their overall classification as low risk.

Although most studies demonstrated a low risk of bias according to the predefined appraisal criteria, the predominance of cross-sectional clinical studies and animal model studies among the included articles limits the ability to draw causative inferences. Because we found repeated associations between elevated levels of TNF-α, IL-1β, and IL-6 and TMJ inflammation, pain, and structural degeneration, consistency across the studies was considered to be generally acceptable. However, inconsistency in sampling matrices, cytokine quantification methods (ELISA, PCR, and multiplex assays), and diagnostic criteria for TMDs caused indirectness and reduced comparability between the studies. A major concern was imprecision in data analysis because most studies had a modest sample size, and only a few studies reported formal power analyses [9,17,18,22,23]. Moreover, selective reporting related to incomplete confounder adjustment, unclear blinding, and insufficient disclosure of funding sources in some studies further downgraded the certainty of evidence. Thus, while the biological plausibility of cytokine involvement in TMDs pathophysiology is strongly supported, the certainty of evidence remains moderate, highlighting the need for larger, well-controlled longitudinal studies employing standardized biomarkers and diagnostic protocols.

### 3.2. Data Presentation

The 15 studies included clinical, in vivo, and in vitro research and spanned diverse populations and experimental models (Table 3). Human investigations primarily involved patients with rheumatoid arthritis (RA) or TMDs, while preclinical studies used rodents and domestic pigs as models to reproduce inflammatory joint conditions. The proinflammatory cytokines with a key role included IL-1β, IL-6, IL-17, and TNF-α. These cytokines were found to contribute to pain, inflammation, and pathological bone remodeling. 

In studies on animal models, TMJ inflammation induced using complete Freund’s adjuvant (CFA) or bovine serum albumin (BSA) reliably produced synovitis and cartilage erosion and increased the intra-articular expression of cytokines (IL-1β, IL-6, TNF-α, and vascular endothelial growth factor (VEGF)). The control groups were administered saline. Bone resorption and cartilage degradation were associated with TNF-α overexpression in transgenic mice, and this effect was modestly modulated by ribosomal S6 kinase 2 (RSK2) deficiency. Estrogen-dependent macrophage polarization further aggravated inflammation through enhanced cadherin-11 and inducible nitric oxide synthase (iNOS) expression, highlighting hormonal modulation of cytokine signaling. 

Some biomarker trends were observed in clinical studies. The elevated levels of TNF-α and IL-1β in the SF were associated with pain intensity, restricted motion and mouth opening, crepitus in the TMJ, and condylar erosion. The ratios of TNF/tumor necrosis factor soluble receptor II (TNFsRII) and IL-1β/interleukin-1 receptor antagonist (IL-1Ra) were found to indicate local cytokine imbalance associated with joint destruction. Higher TNF-α and serotonin concentrations in the SF were observed in patients with established RA than in those with early RA. Salivary and serum biomarkers (e.g., IL-4, interferon-gamma (IFN-γ)/IL-4 ratio, and interleukin-8 (IL-18)) were also explored; however, their diagnostic value for TMDs differentiation remained limited. 

Collectively, data from both animal and human studies confirmed that excessive TNF-α-linked cytokine activity and inadequate anti-inflammatory control (low IL-10 and IL-1ra levels) are the mechanisms underlying structural TMJ degeneration and persistent pain (Table 4). Consolidating evidence from these diverse studies strengthens our understanding that TMDs pathophysiology, rather than being considered purely biomechanical dysfunction, is a complex process mediated by cytokine-associated inflammatory mechanisms.

## 4. Discussion

### 4.1. Results in the Context of Other Evidence

#### 4.1.1. Ex Vivo Studies

The ex vivo studies included in this systematic review consistently showed that TNF-α, IL-1β, IL-6, and IL-17 are the key drivers of TMJ inflammation, bone erosion, and chronic pain in experimental models of TMD, thereby establishing a causal link between proinflammatory signaling and structural joint degeneration [2,21,30]. In TNF-α-overexpressing mouse models, sustained TNF-α signaling induces synovial inflammation, activates osteoclastogenesis, and causes subchondral bone resorption and cartilage degradation in the TMJ, confirming joint destruction through a direct TNF-mediated mechanism [26,30]. In these models, TNF-α upregulation is accompanied by a marked increase in the production of downstream cytokines, including IL-1β, IL-6, and IL-17, indicating an intensified inflammatory network rather than an isolated pathway [30]. Moreover, experimental TMJ arthritis induction in large animal and rodent systems similarly causes synovitis, cartilage matrix breakdown, and loss of proteoglycans, together with elevated intra-articular levels of IL-1β, IL-6, and TNF-α; this finding supports the concept that these mediators are sufficient to reproduce TMJ pathology that mimics human osteoarthritis-like (OA-like) and RA-like phenotypes [2,20]. Estrogen-dependent modulation of macrophage polarization within the inflamed TMJ synovium reveals that systemic biological factors influence the local cytokine environment, as estradiol promotes a proinflammatory M1-like macrophage profile, suppresses IL-10 expression, and aggravates joint inflammation [19]. In rodent pain models, persistent TMJ inflammation activates neuroimmune pathways, including P2X7, cathepsin S, and fractalkine (FKN) signaling in trigeminal regions, consistent with central sensitization of nociception [26]. Taken together, data from these animal studies indicate that excessive TNF-α-mediated cytokine activity, supported by high IL-1β, IL-6, and IL-17 expression levels and reduced anti-inflammatory control, can initiate and perpetuate structural TMJ degeneration as well as a chronic pain state, resembling clinical TMDs [2,19,21,26,27,30].

#### 4.1.2. Human Studies

##### RA-Associated TMJ Studies

Consistent with mechanistic findings, human studies show that elevated levels of proinflammatory cytokines in the SF, serum, or saliva correlate with TMJ pain, dysfunction, and radiographic erosion in TMD patients, including those with RA-associated TMJ involvement [17,18,23,29]. Importantly, emerging evidence indicates that cytokine profiles may differ across TMD subtypes. In patients with inflammatory and degenerative joint disorders (e.g., TMJ arthritis, including RA-associated involvement), higher intra-articular concentrations of TNF-α, IL-1β, and IL-6 are consistently associated with joint destruction, crepitus, and progressive structural changes, supporting their role as biomarkers of disease activity and joint degeneration [9,17,18,23]. Furthermore, clinical cohort-based studies reveal that an increased ratio of TNF-to-soluble TNF receptor in the TMJ is associated with a greater degree of mechanical pain during mandibular function, suggesting that insufficient local antagonism of TNF facilitates uncontrolled inflammation and sensitization [18,29]. Higher intra-articular concentrations of IL-1β and IL-6 are related to more severe TMJ arthritis, impaired mandibular range of motion, crepitus, and progressive structural changes, supporting their role as candidate biomarkers for disease activity and joint degeneration in TMDs [9,17,23]. In addition to that it has been documented that in myogenous TMDs subtype, elevated IL-6, IL-10 and TNF-α are connected with muscle tenderness and fatigue rather than structural joint damage, suggesting a predominant role in peripheral and central nociceptive modulation [30]. While this review addresses TMDs as a broad clinical entity, it should be noted that the strongest evidence regarding cytokine involvement is derived from studies focusing on inflammatory and degenerative TMJ conditions, including arthritis-related disorders. 

##### Human TMJ Studies

Longitudinal and cross-sectional studies further suggest the role of cytokine imbalance in the chronicity of TMJ involvement, where the presence of compensatory anti-inflammatory mediators such as IL-10 and IL-1 receptor antagonist fails to counter persistent TNF-, IL-1β-, and IL-6-mediated inflammation [9,17,18,20,23,24,26,30,31,32,33,34,35,36]. Additionally, serotonin levels in the TMJ SF and circulating inflammatory mediators, including chemokines such as C-C motif chemokine ligand 2 (CCL2), C-X-C motif chemokine ligand 9 (CXCL9), C-X-C motif chemokine ligand 10 (CXCL10), and regulated on activation, normal T-cell expressed and secreted (RANTES), have been implicated in longer disease duration, ongoing pain, and altered occlusal relationships, indicating interplay between inflammatory and neurochemical signaling in symptom maintenance [17,23]. Taken together, evidence from human studies confirms that TMDs are not solely a mechanical disorder of occlusion or joint loading, but they mirror a cytokine-mediated inflammatory condition in which TNF-α, IL-1β, and IL-6 contribute to tissue destruction and pain; the impaired endogenous regulation of these pathways may help explain why some patients develop erosive, debilitating TMJ disease [9,17,18,20,23,24,26,29,30,31,32,33,34,35,36].

### 4.2. Limitations of the Evidence

The evidence synthesized in this systematic review has several limitations that influence the strength, comparability, and generalizability of our findings. The number of eligible studies was relatively small, and most of the studies had modest sample size, which reduced statistical power and limited the ability to detect subtle but clinically relevant associations between cytokine levels and TMDs severity. The studies also exhibited considerable heterogeneity in design, participant characteristics, diagnostic criteria for TMDs, biological sampling sites (SF, serum, and saliva), and analytical methods (ELISA, PCR, and multiplex assays). These variations in methodologies complicate direct comparisons, making it difficult to establish consistent reference ranges or biomarker thresholds. Many studies also lacked standardized control groups or failed to adequately match participants by age, sex, or comorbid conditions; this deficiency could confound the interpretation of cytokine levels. Moreover, while deriving conclusions, several studies did not fully consider systemic inflammatory diseases, such as RA and autoimmune disorders, which can elevate cytokine levels independent of TMDs pathology. Another noteworthy concern is the predominance of cross-sectional and observational study designs, which prevent causal inference, as these studies cannot adequately determine whether elevated cytokine levels promote TMJ inflammation and pain or are an aftereffect of this pathological condition. Additionally, incomplete reporting of confounders, small effect sizes [23], and variations in assay sensitivity introduce potential measurement bias. Some studies did not clearly describe blinding procedures [18], funding sources, or conflict-of-interest disclosures [8], raising concerns regarding their transparency and possible selective outcome reporting. The exclusion of non-English publications may have contributed to language-associated bias. The lack of unpublished or grey literature also increases the risk of publication bias toward studies with positive findings. Finally, the limited amount of longitudinal data restricts our understanding of cytokine production dynamics over time, including changes in cytokine levels during disease progression or treatment response. Collectively, these limitations highlight the need for larger, high-quality, and standardized studies to confirm the biological and clinical significance of proinflammatory cytokines in TMDs pathogenesis and to validate their application as reliable diagnostic or prognostic biomarkers. A meta-analysis could not be conducted because of the heterogeneity of the articles included in the systematic review.

### 4.3. Limitations of the Review Process

Despite adherence to PRISMA 2020 guidelines and registration in PROSPERO, this review has several methodological constraints that should be acknowledged. First, the search was limited to English language publications (published between January 2014 and September 2025) and to four databases, possibly introducing language- and database-associated bias and omitting earlier or non-indexed relevant studies. Gray literature was also excluded, increasing the likelihood of publication bias. Second, screening, data extraction, and quality assessment were conducted independently by multiple authors using consensus procedures; however, the possibility of residual selection and author bias remains. Third, substantial heterogeneity in TMDs diagnostic frameworks, sampling matrices, and assay methodologies complicated the identification of eligible studies and increased misclassification risk at the eligibility stage. Fourth, regarding study design, the included evidence was predominantly observational and often lacked standardized reporting of key confounders such as comorbid pain conditions, medication use, circadian timing of sampling, and menstrual or hormonal status. This reduced the feasibility of conducting a meta-analysis, increasing vulnerability to outcome reporting bias. Fifth, the review relied on published aggregate results without access to individual participant data, which restricted opportunities for harmonized reanalysis and robust covariate adjustment. Lastly, although the review was based on a predefined protocol, minor operational deviations, such as iterative refinement of search terms and clarifications of eligibility criteria, may have occurred, possibly affecting the reproducibility of results. Taken together, these limitations reduce the overall certainty of the synthesized findings and emphasize the need for standardized methodologies and improved data sharing in future research.

### 4.4. Implications for Practice, Policy, and Future Research

The findings of this systematic review support the central role of proinflammatory cytokines, particularly IL-1β, IL-6, and TNF-α, in the pathophysiology of TMDs. From a clinical perspective, measuring cytokine levels in biological fluids, such as synovial fluid, saliva, or serum, can serve as an adjunctive diagnostic and prognostic tool. Incorporating biomarker assessment into standard clinical protocols could facilitate earlier identification of inflammatory TMDs subtypes, enabling more targeted, personalized interventions. For instance, elevated IL-6 or TNF-α levels may help differentiate inflammatory arthropathies involving the TMJ from non-inflammatory myofascial pain syndromes, guiding clinicians toward appropriate anti-inflammatory or immunomodulatory therapies. From a public health perspective, this systematic review underscores the fundamental importance of integrating biomarker research into strategies for the prevention, diagnosis, and management of chronic orofacial pain. These mediators not only contribute to the initiation and enhancement of local inflammation within the TMJ but also participate in inducing persistent chronic pain and tissue degeneration. Future research should focus on several aspects that require prompt attention. First, longitudinal studies are required to clarify the temporal relationships between variations in cytokine levels and disease progression and to establish causality rather than mere association. Second, large-scale, multicenter trials should adopt standardized sampling methods, assay protocols, and diagnostic criteria to reduce interstudy heterogeneity. Third, emerging omics technologies, such as transcriptomics, proteomics, and metabolomics, should be utilized to construct comprehensive inflammatory profiles of TMDs subtypes. From a therapeutic perspective, the involvement of proinflammatory cytokines in TMDs provides a rationale for targeted anti-inflammatory interventions. Intra-articular corticosteroid injections have been shown to provide temporary relief of TMJ pain and dysfunction, particularly in patients with inflammatory joint involvement such as rheumatoid arthritis, likely through suppression of local cytokine production [17]. However, the short-term nature of these effects (in Kroese et al. research the effect lasted for 3 weeks) highlights the need for more sustained therapeutic approaches. In this context, monoclonal antibodies targeting key cytokines, including TNF-α and IL-1β, may represent a promising strategy in some cases, given their established efficacy in systemic inflammatory conditions such as rheumatoid arthritis. Although their application in TMDs remains limited and requires further investigation, their mechanistic relevance suggests potential utility, particularly in patients with pronounced inflammatory phenotypes. Overall, this systematic review emphasizes the utilization of both biomechanical and molecular approaches to better understand and manage TMDs. Bridging basic immunological research with clinical practice has the potential to transform current diagnostic paradigms, optimize treatment selection, and ultimately improve patient outcomes.

## 5. Conclusions

This systematic review describes the pivotal role of proinflammatory cytokines in the development and progression of some TMDs. Evidence from both experimental and clinical studies demonstrates that cytokines, including TNF-α, IL-1β, IL-6, and IL-17, are the central mediators of synovial inflammation, cartilage degradation, bone resorption, and chronic pain within the TMJ. These findings suggest that clinicians should view certain TMDs as disorders with an immunoinflammatory component and not merely as a condition associated with mechanical or functional disruption of the TMJ. The measurement of cytokine levels in the SF, serum, or saliva may enable recognition of inflammatory subtypes of TMDs and facilitate earlier diagnosis, disease stratification, and targeted treatment.

## Figures and Tables

**Table 1:** Search syntax used for identifying relevant publications.

Source	Search Term	n
PubMed	(“temporomandibular disorders”[MeSH Terms] OR “temporomandibular disorder”[Title/Abstract] OR “TMJ”[Title/Abstract] OR “temporomandibular joint”[Title/Abstract]) AND (“cytokines”[MeSH Terms] OR “proinflammatory cytokines”[Title/Abstract] OR “inflammatory markers”[Title/Abstract] OR “interleukin-1”[Title/Abstract] OR “IL-1β”[Title/Abstract] OR “interleukin-6”[Title/Abstract] OR “IL-6”[Title/Abstract] OR “tumor necrosis factor-alpha”[Title/Abstract] OR “TNF-α”[Title/Abstract])	375
Embase	(‘temporomandibular disorder’/exp OR ‘temporomandibular disorder’: ti,ab OR ‘temporomandibular joint’: ti,ab OR TMJ: ti,ab) AND (‘cytokine’/exp OR ‘proinflammatory cytokine’: ti,ab OR ‘inflammatory marker’: ti,ab OR ‘interleukin 1’: ti,ab OR ‘il 1β’: ti,ab OR ‘interleukin 6’: ti,ab OR ‘il 6’: ti,ab OR ‘il6’: ti,ab OR ‘tumor necrosis factor alpha’: ti,ab OR ‘tnf α’: ti,ab OR ‘tnfa’: ti,ab)	690
Scopus	(TITLE-ABS-KEY(“temporomandibular disorders” OR “temporomandibular disorder” OR TMJ OR “temporomandibular joint”)) AND (TITLE-ABS-KEY(“cytokines” OR “proinflammatory cytokines” OR “inflammatory markers” OR “interleukin-1” OR “IL-1β” OR “IL1B” OR “interleukin-6” OR “IL-6” OR “IL6” OR “tumor necrosis factor-alpha” OR “TNF-α” OR “TNFA”))	577
Cochrane Library	(“temporomandibular disorder” OR “temporomandibular disorders” OR “temporomandibular joint” OR TMJ) AND (“cytokines” OR “proinflammatory cytokines” OR “inflammatory markers” OR “interleukin-1” OR “IL-1β” OR “IL1B” OR “interleukin-6” OR “IL-6” OR “IL6” OR “tumor necrosis factor-alpha” OR “TNF-α” OR “TNFA”)	82

**Table 2:** Results of the quality appraisal based on the quality checklist.

	A	B	C	D	E	F	G	H	I	Total	Risk
Dozet et al. (2025) [8]	1	1	1	1	1	0	1	1	0	7	Low
Naujokat et al. (2019) [2]	1	1	1	1	1	0	1	1	1	8	Low
Alstergren et al. (2018) [9]	1	1	1	1	1	1	1	1	1	9	Low
Kroese et al. (2021) [17]	1	1	1	1	1	1	1	1	1	9	Low
Ahmed et al. (2015) [18]	1	1	1	1	1	1	1	1	1	9	Low
Kou et al. (2015) [19]	1	1	1	1	1	0	1	1	1	8	Low
McIlwrath et al. (2017) [20]	1	1	1	1	1	0	1	1	1	8	Low
Ananias et al. (2023) [21]	1	1	1	1	1	0	1	1	1	8	Low
Park et al. (2014) [22]	1	1	0	1	1	1	1	1	1	8	Low
Kroese et al. (2020) [23]	1	1	1	1	1	1	1	1	1	9	Low
Andreev et al. (2025) [24]	1	1	1	1	1	0	1	1	1	8	Low
Georgi et al., 2025 [26]	1	1	1	1	1	1	1	1	1	9	Low
Bonfante et al. (2018) [27]	1	1	1	1	1	0	1	1	1	8	Low
Kurtoglu et al. (2016) [28]	1	1	1	1	1	0	1	1	1	8	Low
Ahmed et al. (2015) [29]	1	1	1	1	1	1	1	1	1	9	Low

**Table 3:** Overview of TMJ research: Study aims and methodologies in humans and animal models.

Author and Year	Country	Study Type	Study Overview
Dozet et al., 2025 [8]	Croatia	Observational, cross-sectional study	Relationship between salivary and serum levels of IL-4, IL-18, and IFN-γ in patients with rheumatoid arthritis (RA), with or without TMD.
Naujokat et al., 2019 [2]	Germany	In vivo study	Five domestic pigs were systemically immunized with bovine serum albumin (BSA) and administered intra-TMJ injections of BSA to induce arthritis; saline injections served as controls. After 10 weeks, TMJs and adjacent tissues were harvested for histological analysis and cytokine quantification to validate the model.
Alstergren et al., 2018 [9]	Sweden	Original research	Assessment of TMJ pain, function, joint sounds, and occlusal changes; SF was sampled (push-pull, hydroxycobalamin method) and analyzed for TNF, TNFsRII, IL-1β, IL-1ra, IL-1sRII, IL-6, and serotonin levels; joints were classified arthritic if any mediator exceeded the normal level; clinical variables were tested (logistic regression models/receiver operating characteristic [ROC] curve) to obtain criteria for possible, probable, and definite TMJ arthritis and to indicate inflammatory activity.
Kroese et al., 2021 [17]	Sweden	Cohort Study	Thirty-five RA patients (median age: 54, 89% females) underwent baseline clinical assessments for maximum mouth opening, TMJ pain at rest, degree of anterior open bite (AOB), and presence of crepitus as well as blood tests for rheumatoid factor (RF), erythrocyte sedimentation rate (ESR), C-reactive protein (CRP), serotonin, and IL-1β. Across 70 TMJs, 53 TMJs received methylprednisolone (with lidocaine) injections at baseline (T0). Clinical and laboratory evaluations were repeated after 3.1 weeks (T1) and again after approximately 6.3 weeks (T2) in 21 patients, 20 of whom received a second injection at T1.
Ahmed et al., 2015 [18]	Sweden	Observational, cross-sectional study	Assess whether the balance or imbalance between proinflammatory TNF and its anti-inflammatory soluble receptor (TNFsRII) in the TMJ SF affects joint pain, and how this interaction influences anti-citrullinated peptide antibody (ACPA) levels. TMJ pain was evaluated under various conditions (at rest, opening, chewing, and palpation), along with mandibular mobility and AOB. SF and blood samples were quantified for TNF, TNFsRII, and anti-citrullinated peptide antibodies (ACPA). Systemic disease activity was also measured using the DAS28 score for RA.
Kou et al., 2015 [19]	China	In vivo study	Adult female rats were ovariectomized to eliminate endogenous estrogen and subsequently administered escalating doses of 17β-estradiol for 10 days. TMJ inflammation was induced by intra-articular injection of CFA. Macrophage polarization and cadherin-11 expression in the inflamed synovium were evaluated at 24 h, with and without the use of antagonists to block cadherin-11 or estrogen receptors.
McIlwrath et al., 2017 [20]	USA	In vivo study	The role of TNF receptor deficiency in chronic orofacial pain was investigated using a “double-hit” inflammatory model (CFA into the TMJ followed by application of colonic mustard oil after 3 weeks). Wild-type and TNFR1/R2^−/−^ mice were compared to assess behavioral hypersensitivity, serum cytokine/chemokine profiles, and responses to pharmacological interventions. The study aimed to model chronic TMJ inflammation and identify alternative molecular pathways that sustain hypersensitivity in the absence of TNF-α signaling.
Ananias et al., 2023 [21]	Brazil	In vivo study	The study compared three induction models of arthritis in rat TMJs using CFA + type II bovine collagen (CII). Twenty-four male Wistar rats were divided into four groups and administered CFA + CII either intra-articularly into the TMJ, intradermally at the base of the tail, or both. After 23 days, TMJs were harvested for histomorphometry evaluation (cartilage thickness, morphology, and collagen/proteoglycan integrity) and cytokine analysis (IL-1β, IL-6, TNF-α, and IL-10).
Park et al., 2014 [22]	Republic of Korea	In vitro and in vivo study	The study evaluated tRORγt-TMD, a cell-penetrating, nucleus-deliverable form of the transcription modulation domain of RORγt. The aim was to specifically inhibit RORγt-mediated transcription and TH17 differentiation without affecting TH1, TH2, or Treg pathways. Delivery efficiency, specificity, and effects were tested in vitro and in vivo. The efficacy of tRORγt-TMD to anti-IL-17 monoclonal antibody and methotrexate (for RA model) was compared.
Kroese et al., 2020 [23]	Amsterdam	Clinical, cross-sectional study	The study assessed the levels of inflammatory mediators (TNF, IL-1β, IL-1ra, IL-1sRII, TNFsRII, and serotonin) in the SF of the TMJ and in blood from RA patients and examined their relationship with disease duration and TMJ-related symptoms.
Georgi et al., 2025 [26]	Germany	In vivo	This experimental mouse study investigated whether Rsk2, a kinase that protects long bones from inflammation-induced damage, also mitigates TNF-α-driven bone loss in craniofacial skeleton and TMJ. By using micro-CT assay, geometric morphometrics, and histological analysis, wild-type, Rsk2-deficient, TNF-α-overexpressing, and double-mutant mice were compared to assess bone morphology, cartilage integrity, and synovial inflammation.
Bonfante et al., 2018 [27]	Brazil	In vivo	Explored molecular mediators of persistent TMJ pain in rats, focusing on neuroinflammatory biomarkers within the P2X7–cathepsin S–fractalkine (FKN) signaling pathway.
Ahmed et al., 2015 [29]	Sweden	Clinical, cross-sectional study	Investigated the relationship between TNF, IL-1β, their endogenous inhibitors (TNFsRII, IL-1ra, and IL-1sRII, and ACPA) in the TMJ SF and serum of RA patients and their associations with MRI-detected bone erosions.
Andreev et al., 2025 [24]	Germany	In vivo	Investigated molecular and cellular mechanisms of TNF-α-mediated TMJ erosion compared with the ankle joint, combining micro-CT assay, histological assay, flow cytometry, qPCR, and bulk RNA-seq to map inflammatory and osteoclastic pathways.
Kurtoglu et al., 2016 [28]	Turkey	Cross-sectional study	Assessed the prevalence and characteristics of TMD in 54 rheumatoid arthritis patients using RDC/TMD criteria and panoramic radiography; examined associations between TMJ findings, RA duration, treatment type, and pain-related variables.

**Table 4:** A comprehensive summary of the primary outcomes from the 15 included studies.

Author and Year	Study Groups	Main Outcomes
Dozet et al., 2025 [8]	RA group (n = 30): patients with RA with/without TMDs Control group (n = 30): patients without RA with/without TMDs	The RA group was compared with the Control group. RA patients exhibited significantly higher salivary IL-4 levels (*p* < 0.001; r = 0.590), significantly elevated serum IFN-γ/IL-4 ratio (*p* < 0.001; r = 0.519), significantly lower serum calcium levels (*p* = 0.002; r = 0.402), and higher GGT levels (*p* = 0.015; r = 0.315). In the comparison of RA with TMD vs. RA without TMD. no specific salivary or serum biomarker differentiated RA patients with TMD from those without TMD.
Naujokat et al., 2019 [2]	AIA (BSA-injected) TMJs in immunized pigs, compared with control TMJs injected with saline (n = 5 animals).	Severe synovial inflammation, loss of cartilage glycosaminoglycans, cartilage surface/disc alterations, and chondrocyte clustering in AIA joints. Elevated levels of synovial cytokines (IL-1β, IL-6, TNF-α, and vascular endothelial growth factor (VEGF)) in AIA vs. control. This porcine TMJ arthritis model is reliable and can be used to study disease mechanisms and evaluate potential therapies.
Per Alstergren et al., 2018 [9]	219 TMJs from 156 individuals: 141 patients with disorders potentially involving TMJ arthritis plus 15 healthy controls (age/sex distribution reported), all examined clinically with SF reference testing.	According to the reference standard, 71% of TMJs were arthritic. Among these, 93% were painful, while 66% of non-arthritic TMJs also showed some pain. Clinical predictors of arthritis included resting pain intensity, pain during maximum mouth opening, number of painful jaw movements, and contralateral laterotrusion (area under the curve [AUC] ≈ 0.72, *p* < 0.001). Arthritic TMJs with higher inflammatory activity, as indicated by synovial mediators, were associated with greater pain during maximum mouth opening and more painful mandibular movements.
Kroese et al., 2021 [17]	Injected joints: 53 TMJs received methylprednisolone at baseline. Follow-up assessments: all patients were evaluated at T1; a subset of patients (21 patients, 29 joints) was followed up at T2, with most receiving a second injection. The study used within-patient comparison across time points rather than between-group controls.	No biomarker correlations: changes in clinical outcomes (mouth opening or pain) did not correlate with ESR, CRP, serotonin, or IL-1β levels. Crepitus resolution: Among joints with initial crepitus (n = 19), 8 joints showed resolution at T1.
Ahmed et al., 2015 [18]	A single cohort of 26 consecutive RA patients with TMJ involvement was studied, without separate control groups, and comparisons were made internally based on SF biomarker ratios and their associations with pain and antibody profiles.	A high synovial TNF-to-TNFsRII ratio was significantly associated with TMJ pain on posterior palpation during maximal mouth opening, suggesting that insufficient local anti-inflammatory regulation enhances mechanical pain sensitivity ACPA levels showed a significant correlation with TNF concentration, but not with TNFsRII concentration, indicating that elevated inflammatory activity is affected more by inadequate anti-inflammatory compensation than by elevated TNF production alone Conclusion: TMJ pain in RA patients is influenced by impaired local cytokine regulation, particularly a relative deficiency of soluble TNF receptor, leading to intense inflammatory and mechanical nociceptive responses.
Kou et al., 2015 [19]	In vivo groups: ovariectomized rats receiving varying doses of estradiol before CFA-induced TMJ inflammation. Subsets with blocking interventions: cadherin-11 antibody or estrogen receptor antagonist (ICI 182,780). In vitro groups (NR8383 macrophages): Treated with TNF-α and with or without estradiol. Co-treated with cadherin-11 blockade or estrogen receptor antagonist	Estradiol significantly enhanced synovial infiltration by proinflammatory M1-like macrophages, as evidenced by the increased presence of CD68^+^ iNOS^+^ cells; M2-like macrophages were scarce. Estradiol dose-dependently upregulated iNOS (M1 marker) while repressing IL-10 and arginase (M2 markers) in NR8383 cells. Moreover, estradiol enhanced cadherin-11 expression, particularly in M1-like macrophages in the inflamed TMJ. Cadherin-11 blockade mitigated estradiol-mediated M1 polarization and TMJ inflammation both in vivo and in vitro; it reversed TNF-α- and estradiol-induced iNOS expression and nitric oxide release, without affecting IL-10 levels.
McIlwrath et al., 2017 [20]	TNFR1/R2^−/−^ mice (genetic deletion of both TNF-α receptors) and wild type (WT) controls. Both sexes included (although cytokine analyses were mainly performed in male mice). Approximately 60 mice were tested across groups	A “double-hit” inflammatory model (TMJ insult with CFA + subsequent colonic mustard oil insult) produced chronic mechanical and heat hypersensitivity persisting for ≥18 weeks, but only in TNFR1/R2^−/−^ mice; WT mice showed full recovery. Serum cytokine/chemokine analysis revealed elevated levels of proinflammatory mediators (CCL2, CXCL9, CXCL10, RANTES, and TNF-α) and decreased levels of anti-inflammatory cytokines (IL-1ra, IL-4, and G-CSF) in TNFR1/R2^−/−^ mice.
Ananias et al., 2023 [21]	G1 (Sham): 0.9% NaCl injected at the base of tail + intra-articular injection in the TMJ. G2 (osteoarthritis [OA] model): Intra-articular administration of CFA+CII in both TMJs. G3 (RA+OA model): CFA+CII injected at the base of tail + intra-articular administration of CFA+CII in TMJs. G4 (RA model): CFA+CII injected at the base of tail. (n = 6 animals per group)	Cytokine profile: IL-6: Highest in G2 (OA) and increased in G3 and G4 vs. G1. IL-1β: Elevated in G2, G3, and G4, with highest levels in G3 (RA+OA). TNF-α: Significantly increased in G3; moderately increased in G2 and G4 vs. G1. IL-10: Elevated in G2 alone; reduced in G3 and G4 vs. G1 Histomorphometry: G2: Increased total cartilage thickness, thickened fibrous/proliferative layers, and disorganized chondrocytes, but higher IL-10 levels (protective/early OA profile). G3 and G4: Reduced cartilage thickness, loss of proteoglycans, collagen fiber disorganization, and chondrocyte apoptosis → features indicating RA-like advanced degeneration. Conclusion: Intra-articular CFA+CII (G2) mimics early OA changes in the TMJ. Tail injections (G3 and G4) induced inflammation/degeneration compatible with chronic RA-like pathology. Combining systemic + local (G3) did not yield additive effects beyond RA alone
Park T. et al., 2014 [22]	In vitro: Mouse naïve CD4^+^CD25^−^CD62L^high^ T cells under TH1-, TH2-, TH17-, and Treg-polarizing conditions. HEK293 cells transfected with RORγt or RORα1 constructs. Splenocytes from C57BL/6 mice. In vivo (mice): EAE-induced mice: treated with PBS, tRORγt-TMD, or anti-IL-17 mAb (both preventive and therapeutic regimens). CIA-induced mice: treated with PBS, methotrexate, or tTbet-TMD (for comparing TH1-targeting). Outcomes measured: cytokine secretion, T-cell subset differentiation, luciferase reporter assays, flow cytometry, and histopathological assays (spinal cord lesions, demyelination, and cell infiltration).	Specificity: tRORγt-TMD efficiently entered the nucleus and inhibited RORγt-driven IL-17 promoter activity, but did not affect RORα1-mediated transcription. TH17 inhibition: Strong suppression of TH17 differentiation and IL-17A/F production, without affecting TH1 (IFN-γ), TH2 (IL-4), or Treg (IL-10). Prevented Treg conversion to TH17 under inflammatory conditions. Gene expression: Downregulated TH17-related genes (IL-21, CCL2, CCL20, IL-12Rβ1, and TLR-4). In vivo efficacy: In EAE mice, tRORγt-TMD reduced disease incidence and severity, both preventively and therapeutically, and outperformed anti-IL-17 mAb in some measures. It also reduced infiltration of CD4^+^ IL-17^+^ T cells into the CNS and decreased spinal cord demyelination. In CIA mice, tTbet-TMD (an analogous TH1 construct) alleviated arthritis, with efficacy comparable to that of methotrexate. Conclusion: Interactomic inhibition of transcription factors (tRORγt-TMD and tTbet-TMD) is a novel, targeted strategy for modulating TH17/TH1-mediated autoimmunity
Georgi et al., 2025 [26]	Four mouse genotypes (n = 4 per group; all males, 10 weeks old): 1. Control (WT): normal mice. 2. Rsk2^−/y^: Rsk2 knockout mice. 3. hTNFα-tg: transgenic mice overexpressing human TNF-α (arthritis model). 4. hTNFα-tg;Rsk2^−/y^: double mutants lacking Rsk2 with TNF-α overexpression.	TNF-α overexpression induced marked TMJ and craniofacial bone loss with condylar erosion and cartilage degeneration. Rsk2 deficiency alone had minimal effects. Although combined TNF-α overexpression and Rsk2 loss did not worsen bone erosion, it increased synovial membrane thickness, indicating enhanced inflammation. Overall, Rsk2 showed only weak protection against TNF-α-induced craniofacial bone damage.
Bonfante et al., 2018 [27]	Male Wistar rats (n = 6/group): controls, acute inflammation (single mBSA injection), and persistent inflammation (three mBSA injections).	Persistent TMJ inflammation increased local IL-12 and IL-18 levels and upregulated the expression of P2X7 receptor, cathepsin S, and FKN in the trigeminal subnucleus caudalis, indicating microglial activation and central sensitization associated with chronic pain. Acute changes in the model were transient.
Kroese et al., 2020 [23]	68 RA patients (80 TMJs) were assigned to two groups according to disease duration: early RA (≤2 years) and established RA (>2 years).	TNF and IL-1β were detected in 24% and 16% of SF samples, respectively. Established RA is associated with higher plasma and SF TNF levels, greater TMJ dysfunction, and AOB. Early RA is related to TMJ pain and crepitus but lower TNF levels. The plasma IL-1sRII level correlated with longer general disease duration, while the plasma IL-1ra level showed a negative correlation with TMJ symptom duration. The SF serotonin level increased with disease duration. Findings suggest that TNF, IL-1 pathways, and serotonin are the key biomarkers of progressive TMJ inflammation in RA.
Ahmed et al., 2015 [29]	22 RA outpatients (44 TMJs); all receiving DMARDs, and 27% patients receiving anti-TNF therapy; mean age: 47 ± 9 years, mean RA duration: 6 ± 5 years.	A high TNF/TNFsRII ratio in TMJ SF correlated with condylar erosion scores (r = 0.32, *p* = 0.036). An elevated IL-1ra/TNF ratio was also associated with bone resorption. ACPA-positive patients showed higher TNF/IL-1ra ratios, indicating enhanced inflammatory activity. Anti-TNF medication reduced the IL-1ra/IL-1sRII ratio, thereby restoring cytokine balance. Findings indicate that insufficient control of endogenous TNF promotes TMJ bone and cartilage destruction in RA.
Andreev et al., 2025 [24]	Female hTNF-α transgenic (hTNFtg) mice (n = 11) vs. WT controls (n = 10). TMJ, ankle, and alveolar bone tissues were analyzed for the expression of genes associated with cytokines, chemokines, and bone resorption.	hTNFtg mice developed severe TMJ inflammation, cartilage degradation, and bone erosion resembling human juvenile idiopathic arthritis (JIA) pathology. Both TMJ and ankle joints showed upregulation of TNF-α, IL-1β, IL-6, IL-17, and osteoclast markers (Acp5, Mmp9, Ctsk, and Nfatc1). The TMJ exhibited distinct metabolic and resorption signatures, including Dkk1 (Wnt inhibitor) and Oscar upregulation, and higher osteoclast activity. Ankle joints exhibited stronger T-cell infiltration and higher expression levels of CCL2, CCL5, and CCL20. Overall, TNF-induced inflammation and enhanced osteoclastogenesis are the key biomarkers of TMJ destruction, distinct from the ankle’s immune-cell-dominant response.
Kurtoglu et al., 2016 [28]	Patients categorized based on TMD involvement: none (9.3%), muscular disorders (64.8%), joint disorders (7.4%), and joint + muscular disorders (18.5%). Treatment: anti-TNF (31.5%) vs. DMARD (68.5%).	TMD prevalence was high (90.7%), with muscular disorders most common. TMJ pain, deviation, and joint sounds were frequent findings; condylar resorption occurred in 4 patients. Although patients taking anti-TNF medication had longer RA duration, TMD involvement was not associated with disability, depression, or RA activity.

## Data Availability

No new data were created or analyzed in this study. Data sharing is not applicable to this review article.

## Abbreviations

IL: interleukin; IFN-γ: interferon gamma; TMD: temporomandibular disorder; TMJ: temporomandibular joint; BSA: bovine serum albumin; TNF: tumor necrosis factor; TNFsRII: tumor necrosis factor soluble receptor II; IL-1β: interleukin-1β; IL-1ra: interleukin-1 receptor antagonist; IL-1sRII: interleukin-1 soluble receptor II; IL-6: interleukin-6; RA: rheumatoid arthritis; ROC: receiver operating characteristic; AOB: anterior open bite; RF: rheumatoid factor; ESR: erythrocyte sedimentation rate; CRP: C-reactive protein; ACPA: anti-citrullinated peptide antibody; DAS28: disease activity score 28; CFA: complete Freund’s adjuvant; TNFR1/R2^−/−^: tumor necrosis factor receptor 1 and 2 knockout; CII: type II collagen; OA: osteoarthritis; RORγt: retinoic acid receptor-related orphan receptor gamma t; TH17: Type 17 T helper cell; TH1: Type 1 T helper cell; TH2: Type 2 T helper cell; Treg: regulatory T cell; Rsk2: ribosomal S6 kinase 2; FKN: fractalkine; MRI: magnetic resonance imaging; qPCR: quantitative polymerase chain reaction; RNA-seq: RNA sequencing; GGT: gamma-glutamyl transferase; AIA: antigen-induced arthritis; TNF-α: tumor necrosis factor-alpha; VEGF: vascular endothelial growth factor; AUC: area under the curve; iNOS: inducible nitric oxide synthase; M1: classically activated (proinflammatory) macrophage phenotype; M2: alternatively activated (anti-inflammatory) macrophage phenotype; IL-10: interleukin-10; CCL2: chemokine (C–C motif) ligand 2; CXCL9: chemokine (C–X–C motif) ligand 9; CXCL10: chemokine (C–X–C motif) ligand 10; RANTES: Regulated on Activation Normal T-cell Expressed and Secreted; G-CSF: granulocyte colony-stimulating factor; NaCl: sodium chloride; HEK293: Human Embryonic Kidney 293 cells; RORα1: retinoic acid receptor-related orphan receptor alpha 1; EAE: experimental autoimmune encephalomyelitis; PBS: phosphate-buffered saline; tRORγt-TMD: transducible RORγt transcription modulation domain; mAb: monoclonal antibody; CIA: collagen-induced arthritis; tTbet-TMD: transducible T-bet transcription modulation domain; TLR-4: Toll-like receptor 4; CNS: central nervous system; WT: wild type; hTNFα-tg: human tumor necrosis factor alpha transgenic; P2X7: P2X7 purinergic receptor; SF: synovial fluid; DMARDs: disease-modifying anti-rheumatic drugs; JIA: juvenile idiopathic arthritis; Acp5: acid phosphatase 5 (tartrate resistant); MMP9: matrix metalloproteinase-9; Ctsk: cathepsin K; Nfatc1: nuclear factor of activated T-cells cytoplasmic 1; Dkk1: Dickkopf-1 (Wnt signaling pathway inhibitor); Oscar: osteoclast associated receptor; CCL5: chemokine (C–C motif) ligand 5; CCL20: chemokine (C–C motif) ligand 20.
